# An Energy Efficient Simultaneous-Node Repositioning Algorithm for Mobile Sensor Networks

**DOI:** 10.1155/2014/785305

**Published:** 2014-07-23

**Authors:** Muhammad Amir Khan, Halabi Hasbullah, Babar Nazir, Imran Ali Khan

**Affiliations:** ^1^Department of Computer and Information Sciences, Universiti Teknologi Petronas, 31750 Tronoh, Perak, Malaysia; ^2^Department of Electrical Engineering, COMSATS Institute of Information Technology, University Road, Tobe Campus, Abbottabad 22060, Pakistan; ^3^Computer Science Department, COMSATS Institute of Information Technology, University Road, Tobe Campus, Abbottabad 22060, Pakistan

## Abstract

Recently, wireless sensor network (WSN) applications have seen an increase in interest. In search and rescue, battlefield reconnaissance, and some other such applications, so that a survey of the area of interest can be made collectively, a set of mobile nodes is deployed. Keeping the network nodes connected is vital for WSNs to be effective. The provision of connectivity can be made at the time of startup and can be maintained by carefully coordinating the nodes when they move. However, if a node suddenly fails, the network could be partitioned to cause communication problems. Recently, several methods that use the relocation of nodes for connectivity restoration have been proposed. However, these methods have the tendency to not consider the potential coverage loss in some locations. This paper addresses the concerns of both connectivity and coverage in an integrated way so that this gap can be filled. A novel algorithm for simultaneous-node repositioning is introduced. In this approach, each neighbour of the failed node, one by one, moves in for a certain amount of time to take the place of the failed node, after which it returns to its original location in the network. The effectiveness of this algorithm has been verified by the simulation results.

## 1. Introduction

Wireless sensor networks (WSNs) are one of the most significant technologies today as they have become widely utilized and have come to be a research field that is well-developed. Typically, WSNs are made up of a large set of compactly installed small sensor nodes that are mobile. These nodes are inexpensive and use little power. They are most often effectively applied to monitor various environments, process information and for communicates each other by radio [1–5]. WSNs can lower delay and cost during the time of development. Moreover, they are applicable for use in many types of environment. They are used in situations where deployment of normal wired sensor networks would be impossible. These environments are such as those found in outer space, deep oceans, or battle fields [6, 7]. For the most part, sensor nodes are utilized for monitoring in areas of home, health, and military. Because they act promptly, are self-organised, and have fault tolerance individuality, sensor networks used in military applications, for example, can be extremely applicable to many systems in the armed forces. Such military uses are control, command, surveillance, communication, and targeting. However, when used in the health industry, they are implemented to help disabled patients as well as in monitoring patients. In addition, the management of inventory and monitoring of disaster areas and product quality are examples of other commercial matters that can also use these sensor nodes [8–10].

When these nodes are deployed in unattended environments that are very harsh, the depletion of the onboard energy or some type of physical damage can result in the network being separated into multidisjoint blocks. This can cause it to stop working resulting in the loss of the node. Such areas are commonly known as sensing holes. So that these holes can be filled, extra sensors or sensors that were deployed earlier have to be moved so that these failures can be resolved, or as a response to an event that necessitates moving a sensor to a certain area [11–14]. The internode connectivity is vital for the effectiveness of an application; moreover, there are nodes that have a role to play in keeping the flow of the data from the sensors, which are in place, and from remote users [15].

When a substitution for the dead node needs to be made, it could use up a lot of time and often may not be possible in dangerous environments such as on the battle field. There have been studies that have proposed node repositioning as an efficient tool in the restoration of partitioned networks [16]. During the time that the network is operational, dynamical node repositioning is needed to increase the network's performance. An example of such a situation would be when several sensors surrounding a base-station cease to work because their batteries have been used up; some extra sensors from different areas of the region being monitored can be identified and moved to the affected area for replacement of the nonfunctioning sensors. This will increase the lifetime of the network [17–19]. Moreover, this type of dynamic relocation is quite advantageous in an application that is used to track a mobile target. Some of the sensors, for example, may be moved to a position that is near the target to improve the accuracy of the sensor's data. Furthermore, for safety, some applications could require keeping the base-station a suitable distance away from targets that are dangerous, for example, an enemy tank. This is accomplished by moving it to a less dangerous location so that its availability is guaranteed [20–22].

It is quite difficult to relocate nodes whilst the network is under its normal operating condition. This movement to a new location is made as a reaction to an environment- or network-based event. This is unlike the initial deployment. Further, because the relocation is based on an occurring event, continual monitoring of the state and performance of the network is needed. It also requires an analysis of events taking place in the area of the node [23–25]. Furthermore, this process of relocating the node must be handled with care because of the potential disruption to the delivery of the data. These earlier studies were more focused on restoring broken connectivity with no consideration taken in regard to the unconstructive impact that relocating the nodes could have on the coverage, such as an extra node being relocated in place of the failed node.

Suggesting that the QoS of the sensor network can be measured by coverage with good connectivity [26, 27], therefore, it would appear that connectivity and coverage must both be taken into consideration. It has been shown in an earlier study that failure of an individual node with no redundant node available in the network is similar to baseline methods. This paper proposes an energy efficient simultaneous node repositioning algorithm; moreover, it contributes to filling this hole in the research. Not like other methods that reposition nodes in order to readjust the topology of the network, the algorithm that has been proposed makes an attempt to maintain the network topology as it is and localize the recovery's scope. In general, a node's failure is handled by the replacement of the node, temporarily, with one of its neighbours. These neighbours move to the failed node's location one after another. When a failed node is detected, its neighbours work together to create a schedule so that each of the neighbours knows when to move itself to the location of the failed node. A substitute node will return to its starting position after serving for a certain amount of time. This lets other substitutes for the failed node take its place. This is repeated again and again. As this proposed algorithm is distributed, it creates quite a limited amount of messaging overhead during the process of the recovery.

The main contributions of this paper can be summarized as follows.

- Firstly, a method is proposed to dynamically reposition the nodes when simultaneous nodes failure occurred in the network to improve the performance of the network.
- Secondly, an energy efficient simultaneous nodes relocation algorithm for mobile sensor network is introduced to address the simultaneous nodes failure problem. The proposed algorithm strives to keep most of the network topology intact and localize the scope of recovery. The failure of a node is tolerated by temporarily replacing it with one of its neighbours. These neighbours take turns in moving to the position of the failed node.
- Thirdly and lastly, extensive simulations are carried out to evaluate the performance of the proposed protocol, by comparing its performance with baseline approaches. The results demonstrated that our algorithm has successfully minimized the total distance travelled and has improved the other QoS parameters like number of exchanged messages, average number of nodes moved, and percentage of reduction in field coverage.

The following sections of this paper are organised as follows. In Section 2, related work is summarized. In Section 3, simultaneous node relocation algorithm for mobile sensor networks is proposed. Simulation results are presented in Section 4, and conclusions and future work are offered in Section 5.

## 2. Related Work

Related to this category are three algorithms: the VECtor-based algorithm (VEC), VORonoi-based algorithm (VOR), and Minimax algorithm. They were suggestions from Wang et al. [28]. There is a close relationship to each of these three algorithms with the Voronoi polygon of the sensor node or point. This is where a node or point is located closer than another node or point to the sensory boundary. In a situation such as this, the VECtor-based algorithm is prepared following Coulomb's law as a process of equating that displays the prevention amongst the electrostatic particles. Consequently, the node that is used for the dispossession of an element of a node's Voronoi polygon is moved away from the other nodes that are close with a force that is comparative to the distance it is from the angular points of the polygon or from the nodes themselves. Heo and Varshney [29] proposed a corresponding procedure as an indication of Coulomb's law but in this case no consideration is made to the node's Voronoi polygon. Each node functions, in turn, as a substitute that is going to move far away, proportionally, to the nodes' compactness in its locale usually nearby. It is able to cause the nodes to relocate to the closest neighbouring point of its Voronoi polygon, at the same time, putting forth a polygon that is more regular. In fact, the VORonoi-based algorithm, the nodes in a number of locations to fluctuate. Meanwhile, in the Minimax algorithm, a rather small and common movement can be found. This is true even when there is not as much fluctuation. However, all in all, these algorithms could, in fact, bring forth movements, intermittently, which afterward cause wastage of both time and energy.

In order to reduce how long the course is, a method that is based on a proxy without any sensor node movement. The exception would be in the case that their destination is calculated beforehand [30]. In their work, they focused more on a system that corroborates with both immobile and mobile sensors. In this situation, the mobile nodes are responsible for loading the extent of the positions from which the nodes are absent. This is accomplished in manner that is distributive and can be predicted by immobile nodes. This implies that the movement of the mobile nodes is logical and that the immobile nodes as the agents are in a logical position. Using this method will result in the distance being reduced by quite a bit. The reduction could be from its mean or from the totality in which the mobile nodes are directed along in order to maintain an equal rate of coverage [31]. On the other hand, the result might only be an increase in the message density. In general, the assumption can be made here that methods such as these have a tendency to avoid holes in the coverage rather than actually concentrating on the connectivity. Another solution was proposed by Wu and Yang. Their proposal known as SMART made use of 2D scrutinisation of the selected networks. The aim was to cut down on the overall deployment time [32]. A method adopted from a well-accepted design for a balance of the load amongst nodes in a number of conformations on the parallel processing was used. The process is divided into separate components, implemented on various processors. Subsequently, this design is utilized in multicluster WSNs. In these networks, each single cluster is constituted by a 2D mesh that is formed by square cells. Moreover, several sensors will be added to a single cell to represent the load on a cluster. The location in an interconnection in vertical or horizontal indices as well as the number of sensors in its cluster indicates that a cluster-head's only communication can be with sensors in similar locations in other cells close at hand. In due course, the corresponding coverage that is achieved is related to the issue of balancing the amount of the energy needed to level the distribution of the sensors amongst the clusters. All in all, these methods are more focused on avoiding gaps in the coverage than they are in sustaining connectivity.

Situating nodes into a structure that is very efficient is the aim of the algorithm mentioned above. When the network application begins, a node failing could cause the efficiency to be reduced quite a bit. Moreover, alterations in the needs of the application could also have an influence on the meaning of efficiency itself. In both situations, the nodes have to reposition themselves to maintain a network layout's efficiency. Wang et al. [28] proposed the cascaded movement to replace failed sensor nodes. It achieves this using nearby redundant nodes to repeatedly take the place of the failed node. Some other studies have also taken connectivity into consideration. This is explained in [28] where, for example, one method makes the decision to maintain connectivity of two degrees. It does this, even if a link or node fails, by relocating a subset of the nodes. Whilst the movement of the nodes is comparable to our approach, if the need for 2-connectivity is stressed upon, the application-level functionality might be constrained. However, in large-scale networks, where the nodes' resources are constrained, this may not be practical. In our study, the approach with the closest relation to RIM (recovery through inward motion) found in the literature is DARA (distributed actor recovery algorithm) [33]. In the approach, each of the nodes is required to keep a list of their 2-hop neighbours. They must choose one of the failed node's neighbours to move on the basis of how many communication links there are. However, a set of rules for a scattered actor improvement for fixing the actor networks that have been partitioned was suggested by Akkaya et al. [34]. Real-time restoration is raised by DARA with no hypothesis being made as to how the network is interconnected before an actor malfunctions. Moreover, there is no interrelation to each other. Instead of relocating a block [35], DARA may track a flown reposition of several actors. In this way, a small number of movements are assumed rather than a whole block movement. This leads to the entire movement of the actors being required in the movement or flown reposition. Moreover, the block movement involves being aware of all of the actors in each separated network moving towards the location and how far the movement goes. This introduces extra messaging overhead. In this situation, it is performed so that the two-hop neighbour lists can be created and easily maintained.

Meanwhile, postdeployment connectivity and coverage were considered by Akkaya and Younis [36, 37]. Coverage is increased without breaking any existing internode links when C^2^AP (coverage-aware connectivity-constrained actor) spreads out the connected nodes whilst with COCOLA (connected coverage and latency aware actor placement), a network architecture that is hierarchical is considered. In this approach, nodes on a higher tier are repositioned, incrementally, so that the coverage can be maximised but with no extension to the data route to the node on the first tier. This is so that a desired bound on data latency is maintained. However, the impact that a failed node has is not handled by either C^2^AP or COCOLA.

The idea of assigning a mobility readiness index (MRI) to each actor according to the presently performed task's impact was proposed by Abbasi et al. [38]. It is at this time the value of the MRI that allows an actor to be relocated or not. The network topology is such that if noncritical actors fail, the interactor connectivity providing alternate routes will not be damaged. However, three disadvantages have been noted with their proposed method. The first disadvantage is that C2AM (interactor connectivity with application level constraints on actors' mobility) is a reactive method and, therefore, it might not be suitable for applications that are mission-critical and time-sensitive. The second disadvantage is that C2AM does not take into consideration the actor's ability when relocating the actor during the process of recovering a malfunction. Here, performing the relocation of a noneffective actor might cause the opposite of the effect wanted in the application. The third disadvantage is that C2AM needs 2-hop information to be maintained and does not care about the coverage of the actor.

The NN (nearest neighbour) and RIM (recovery through inward motion) algorithms were in turn proposed by Younis et al. [39]. RIM is a distributed approach for the restoration of connectivity by way of an inward motion. The main concept behind the approach is that upon the failure of node F, its nearest neighbouring nodes will reposition themselves inwards to the location of the failure so that they will have the ability to link with each other. This is a result of the neighbouring nodes referring to the nodes that have been directly impacted on by the failure of node F. Thus, when they can reach each other again, the connectivity of the network is restored to its level before the failure took place. The relocation process is performed in a repeated manner for any node that has failed in order to move one of its neighbour nodes, for example, one of the nodes that has already relocated to the failed node's position. The NN approach, like RIM, follows voracious heuristics. Upon the failure of a node, the NN will travel to its nearest neighbour, FNN, where F is located. It takes this action in order to repair the disrupted connectivity around node F. In response to the relocation of FNN, its closest neighbour from among those nodes near its location will travel to the original position of FNN and settle there. This process will be repeated. When no neighbour can be found for a relocated node as far as the edge of the network or all of the network nodes have already been repositioned, the NN will stop. Unlike RIM which uses a 1-hop neighbour list, NN requires each node to have knowledge of its 2-hop neighbours. Because of this, the closest neighbouring node will be determined ahead of the F node failing. This is where, neither RIM nor NN are concerned with the impact that the restoration of the connectivity has on the coverage of the network. Repositioning permanently like this is avoided by using the algorithm known as coverage conscious connectivity restoration (C^3^R) [22]. Whilst exchanging another node for the neighbour node restores the connectivity, the fact of the matter is that it only changes the gap in coverage to another area of the field. This could be in the inner area of the network or at its outer edge. This could be handled with the temporary replacement of the node that has failed with one or more of its neighbouring nodes.  Table 1 shows comparative summary of discussed state of the art protocols, in terms of some basic parameter.

## 3. Proposed Protocol

### 3.1. Problem Description

**Figure 1:**
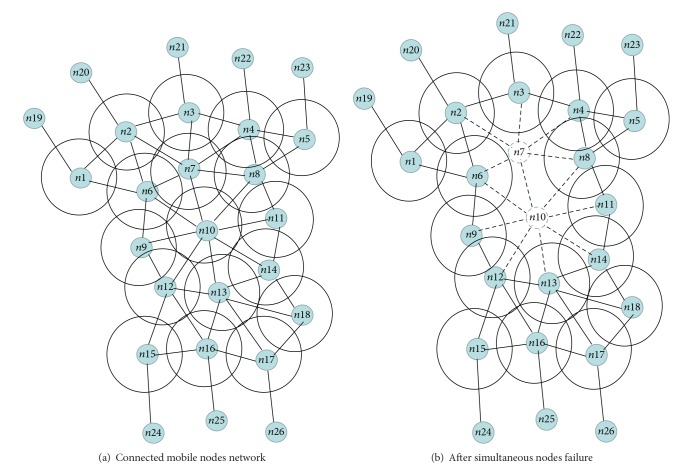
A connected network of mobile node. Individual node denoted the communication range.

Not only could the coverage of the network be affected by losing a node as a result of node failure but also it could have an effect on the connectivity of the network. The proposed method's procedure for restoration of connectivity starts off in the same manner as C^3^R. The focus of this work is on keeping the network connected while maintaining the prefailure coverage level when there is a failure of simultaneous nodes. The proposed method can be referred to in Figure 1.  Figure 1(a) shows the topology of a network that has well connected nodes. As shown in Figure 1(b) nodes 7 and 10 failed simultaneously and all the neighbouring nodes relocate towards nodes* n*7 and* n*10 in order to start recovery process. It is possible that relocating other nodes to replace nodes that have failed can restore connectivity. However, it does not solve the problem as it only moves the gap in the coverage area to another position of the field. This could be in the inner area or at the outer edge of the network. If the failed node is replaced temporarily by one or more of its neighbours, this issue could be dealt with. In other words, when there is a failure of the participating nodes' neighbour, each participating node will make a decision related to some particular criteria as to which neighbour node it will join. The nodes involved will take turns moving back and forth which will result in the topology of the network as well as its coverage being nearly the same as they were before the failure. An energy efficient simultaneous node repositioning method for network connectivity and coverage recovery has been proposed in this paper.

### 3.2. Proposed Solution

As discussed earlier, the network could be partitioned into disjoint segments and/or there could be a gap in the network coverage if node F failed. If the node, A, is repositioned to the failed node's location to facilitate connectivity restoration and node A is not an extra node, then the gap in the coverage is simply moved to another location. As a result, connectivity is still lost because of the relocation of node A. However, if node A could return to its home position after spending only a certain amount of time at node F's spot, a major change in the topology of the network could be avoided. This to and from movement of A restores the network connectivity and coverage from being lost permanently but does not result in a cascading motion. Other close neighbours of node F could do likewise. In this way, the process of recovering connectivity could then be rotated amongst the other nearest neighbours of the failed node. This would continue until there is no further introduction of any new, permanent loss of connectivity and/or coverage. Furthermore, the process used for the recovery of the network should be not only fast but also lightweight. As stated previously, the failure of a node which causes a network to be partitioned is the most challenging problem and it is very serious. In such a situation, when trying to restore connectivity, the main problem is that some nodes could be unreachable by others. As such, it becomes difficult to achieve a well-coordinated noncentralised process of recovery. Further, the sensor nodes, which are resource-constrained, require that the overhead be minimised. A solution would be for all of node F's neighbours to simultaneously begin moving towards the site of node F. Eventually, the nodes would arrive at a position where they would be in range to communicate with each other. At that time, they would synchronise their clocks and decide on a schedule for the recovery plan. A time slot could be assigned to each neighbour as to when each would move to the failed node's area during the recovery process. The schedule would be agreed upon and each node would return to its home location to begin to follow the new schedule and initiate the actual process of recovery.

### 3.3. Explanation of Proposed Protocol

It is obvious that when a network has extra nodes, replacing the failed node F with one of the extra nodes is the best, most effective way to solve the failure problem in terms of the coverage and connectivity of a network. On the other hand, without extra nodes it is impossible to keep degradation from taking place. The philosophy behind the design of the proposed method, as indicated above, is to keep from having to permanently replace a node that has failed. To achieve this end, the role that used to be played by F for sensing and for routing data in the network is carried out by its nearest neighbours instead. However, the neighbours involved in the recovery must still take care of their own tasks. As a result, the jobs that are performed for failure tolerance are functions which add load to these neighbouring nodes.

#### 3.3.1. Process before Node Failure

In our approach, a prefailure list of 1-hop neighbours is the only knowledge required of each node. This list is created before deployment and each node broadcasts a HELLO message as an introduction to its neighbours. Moreover, the nodes must each determine the ID and position of each of its neighbouring nodes. With the proposed approach, only approximate GPS-based coordinates are needed for the nodes' locations. This data is needed for use only in the case of a failing neighbouring node. Moreover, in order to verify that they are available, nodes will transmit heartbeat messages to their neighbours from time to time. Thus, if a node, A, has not received its predetermined count of heartbeat messages from a neighbouring node, F, it will take it to mean that the node, F, has failed. Upon beginning to relocate, a node will transmit a message of its move to each of its neighbours. In this way, it will not be mistakenly believed to have failed. Moreover, this means that the list of the neighbouring nodes could be updated every time the location of one of them is altered.

Node A initiates the process of the recovery as soon as it detects that a neighbour node, F, has failed. It is noteworthy that there are two general strategies available. The first strategy involves determining if the failure of node F will cause the network to be partitioned, and a response will be made only if F is a cut-vertex. This solution, as adopted by DARA, prevents an overreaction from being caused when a failure not preventing the other nodes from communicating with each other takes place. Although, this option requires that there be a procedure for nontrivial cut-vertex detection. This necessitates that 2-hop information be available which causes increased messaging overhead. Furthermore, there is no consideration of the effect that it will have on coverage. The second strategy only implements a process for the recovery when coverage is lost irregardless of node F being a cut-vertex or not. This proposed approach uses this strategy as it does not cause major alterations in the topology of the network when restoring connectivity. Moreover, it deals with the gap caused in the coverage of the network.

#### 3.3.2. Neighbours' Node Management

Node A starts the process of recovery as soon as it detects that its neighbour node F has failed. The first thing that node A must determine is whether or not F has neighbouring nodes which could take part in the recovery process. From here on, node F's neighbours will be known as concerned nodes. Node A will not be aware of all of the concerned nodes since the individual nodes keep a list of only their 1-hop neighbours. These other concerned nodes could also be making plans at the same time for their part in recovering from node F failing. The only way that all of the concerned nodes can coordinate with each other is if each of them were to travel towards the location of node F until they could be positive that they were in the communication range of all of the others. It must be understood that the failure of node F could cause all of the routes of communication among these concerned nodes to be disrupted. However, a node needs only to travel for a distance of rc/2 from node F, where a node's communication range is rc, to be able to connect with each and every one of the concerned nodes. As the concerned nodes might not detect and react simultaneously to node F's failure, some synchronisation is needed for this scenario. In lieu of that, the concerned nodes could all travel to the position of the failed node. Then, the first to get there, say node J, would communicate with the other nodes. At that time, they would synchronise with each other. This would negate the requirement for the synchronisation of all of the nodes with each other to be distributed. However, it could cause the travelling distance to be increased which would lead to an increase in the overhead. Even so, our approach favours this method because of the high degree of coordination involved in the proposed approach. Another reason for this choice is that it is difficult, in practice, to come up with a waiting time that is suitable in order to ensure a meeting time for all of the concerned nodes. From now on, the recovery coordinator or the coordinator of the recovery will refer to node J.

#### 3.3.3. Input Parameter Calculation Values

The recovery coordinator performs the role of synchronising the participating nodes, developing a plan for the recovery and distributing the plan to the nodes involved. Later it will be seen that the nodes that are involved in the recovery process are selected based on the coverage overlap of the nodes, how close they are to the failed node, and how much residual energy they possess. The nodes must each calculate these parameter values as they apply to themselves. Then they share the values with the node coordinating the recovery. The coverage overlap of a particular node is the ratio of the entire area that the node covers and which is situated inside the sensing range of one or several of its neighbours. Determining the overlapped coverage is made simple because the nodes know their location in relation to their neighbours. This is accomplished by basing it on the sensing range, *r*_*s*_, of a node. When a disk coverage model is assumed, the area of the overlap can be calculated by taking into consideration the relationship between the node's closeness to its neighbours and the *r*_*s*_. When the neighbour is closer, there is a larger intersection between the two circles of the radii *r*_*s*_ around the nodes. In addition, the overlapped coverage of a node will be greater when there are more neighbours within the distance *r*_*s*_. Two neighbouring nodes have overlapped coverage if the distance, *d*, between them satisfies

(1) $$\begin{array}{c} d\leq 2\times r_{s}. \end{array}$$

**Figure 2:**
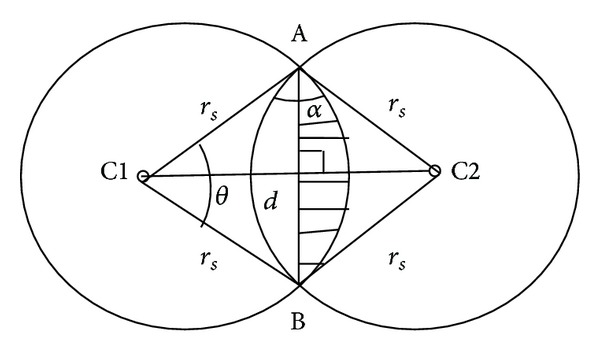
Illustration of the calculation of the overlapped coverage of nodes C1 and C2.

Figure 2 shows that the coverage of the overlap from two nodes is able to be estimated if the area of the chord (*θ*), hereafter referred to as area (*θ*), is found. The distance, *d*, between two nodes, if they are neighbours, can be calculated. When the law of cosines in fundamental Euclidean geometry is used, a node can compute

(2) $$\begin{array}{c} ∠\alpha =2\sin^{-1}\frac{d}{2r_{s}}. \end{array}$$

Then, a triangle with the value of *y* can be calculated by making use of the property of the sum of angles. In Figure 2, the area (*θ*) was the difference between the area of sector AB and triangle ACB. It was calculated as presented as follows:

(3) $$\begin{array}{c} \text{area}\left(\theta \right)=\frac{\theta }{\pi }r_{s}^{2}-\frac{d}{2}\times r_{s}\sin\left(\frac{\theta }{2}\right). \end{array}$$

The formula for the area of a chord, presented below, was utilized to find the overlapping ratio:

(4) $$\begin{array}{c} \text{Overlap}=\frac{2\times \text{area}\left(\theta \right)}{\pi r_{s}^{2}}. \end{array}$$

#### 3.3.4. Recovery Plan Implementation

The basic main factors for designing and implementing the plan for the recovery process are given as follows.

- Participating node A is able to calculate its coverage overlap, distance to F, and energy reserve before it begins to relocate to location F.
- Node A verifies with its neighbouring nodes for temporary relocation in avoiding to declare faulty that neighbour find other route or buffer the data until node A returns to its original position.
- If two nodes claim to be the recovery coordinator, the node closest to F with the lowest ID will be given the task. That node will then broadcast the message to all of the participating nodes.
- This recovery coordinator keeps the list of the ranking which is determined by taking into consideration the coverage overlap, distance travelled, and the amount of energy in reserve. It then uses the round robin manner to set the priority of the relocation.
- During its return trip, A informs its neighbouring nodes and begins the transmission of the buffered data packets again. The same node will repeat a like preset process after that.
- Once it gets below the threshold, the node will transmit a request. The node presently situated in the location of F will receive the request. This node will take the place as the new recovery coordinator and produce a new schedule.

The main factors for designing and implementing the plan for the recovery of connectivity in the case of simultaneous nodes failing at various times are as described below.

**Figure 3:**
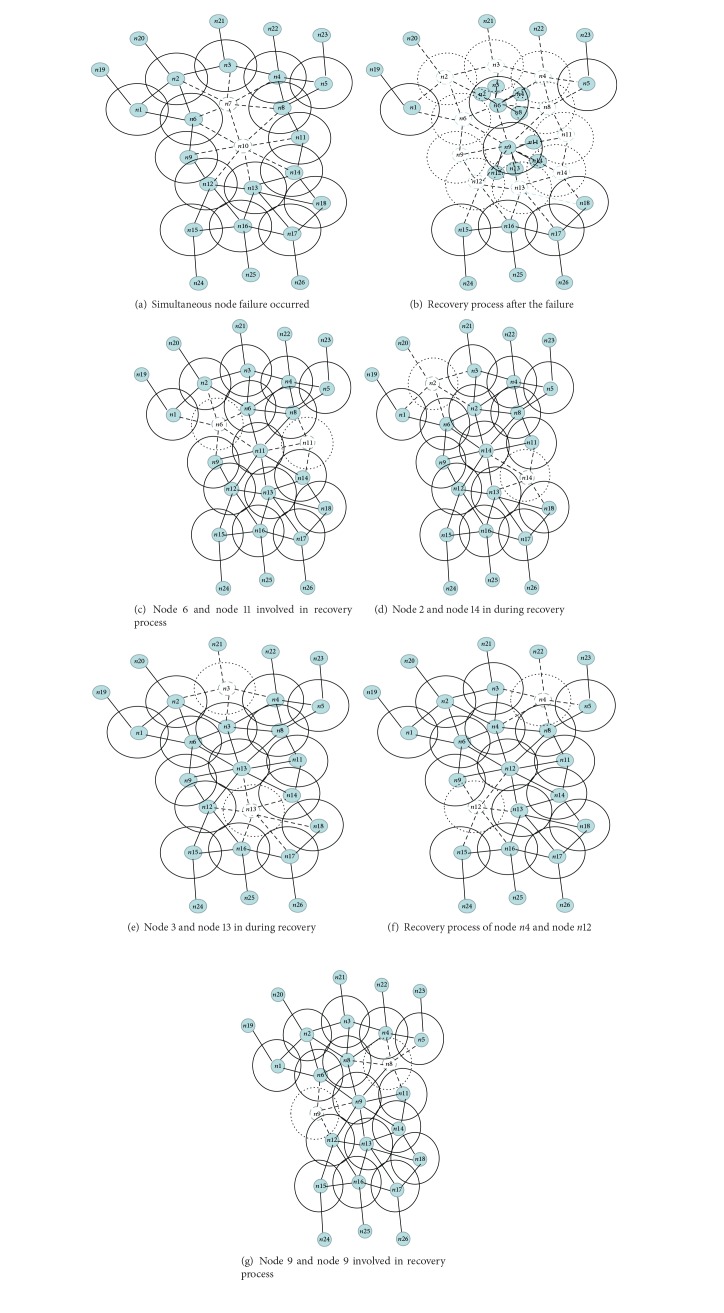
Example that illustrates the operation of proposed technique.

- As shown in the Figure 3(a) node 7 and node 9 simultaneously failed and in order to start recovery process the neighbour of the failed nodes will start the recovery process they move towards the failed nodes.
- Node 6 and node 8 both are in the range of failed nodes n7 and n10. Node 6 and node 8 first calculate their overlap coverage and distance with the failed nodes, as shown in the Figure 3(b). Due to high overlapping with the failed node n7, the node n6 and n8 will move towards the n7 to participate in the recovery process.
- If the overlapped distance is equal then neighbour node will relocate to that neighbour failed node having less node ID.
- According to Figure 3(c) Node 6 and node 11 is the first node in the recovery schedule they will relocate to the failed nodes.
- Figure 3(d) shows that after relocation node 6 and node 11 are back to their initial position. Node 2 and node 14 will relocate to the failed nodes.
- In Figures 3(e)–3(g) after relocating back to their initial positions according to recovery schedule the rest of the nodes will relocate in round robin fashion.

Algorithm 1, explains the recovery process when simultaneous node failure occurs.

## 4. Simulations and Results

The simulations were carried out based on the developed system model using OMNet++ [40] for evaluation of how the proposed approach performed. A comparison is made against two contemporary protocols: RIM and NN [39]. The details of the setup of the simulation, the energy model, and the discussion of the results are presented in this section.

### 4.1. Simulation Setup

The experimentation involved in the simulation process consisted of WSN topologies that were randomly produced with nodes in different numbers as well as different ranges of communication. The number of nodes was set to 25, 50, 75, 100, and 125 in a field of 1000 × 1000 m^2^. RIM and NN make no accommodation for varying communication and sensing ranges so the values of *r*_*s*_ and *r*_*c*_ were maintained as equal throughout all of the experimentation. On the other hand, the experimentation for the proposed approach was carried out by using different sensing and communication ranges as well as by measuring the alterations in the coverage field. These ranges were set to 25, 50, 75, 100, 125, and 150 m. Each node started with an energy level of 100 J. The energy used for communicating, sensing, and moving was calculated on the basis of the specified model.

### 4.2. Sensor's Energy Model

For the simulation, the models for energy consumption for different activities of a sensor node are summarised below.

*Communication Energy Dissipation.* In this model, the main energy parameters for communication were the energy/bit used by the transmitter electronics (*β*_11_), energy used up in the transmit op-amp (*β*_2_), and energy/bit used by the receiver electronics (*β*_12_). If a 1/dn path loss was assumed, the consumed energy was

(5) $$\begin{array}{c} E_{tx}=\langle \beta_{11}+\beta_{2}d^{n}\rangle \times s,\\ E_{rx}=\beta_{12}\times s, \end{array}$$

where *E*_*tx*_ was the energy used to send *s* bits and *E*_*rx*_ was the energy used to receive *s* bits.  Table 2 specifies the energy parameters.

*Sensing Energy Dissipation.* The energy required to sense one bit was assumed to be a constant (*β*_3_) where the total energy used up to sense *s* bits was

(6) $$\begin{array}{c} E_{\text{sensing}}=\beta_{3}\times s. \end{array}$$

In the simulation, *β*_3_ was equal to 25 nJ/bits. Motion was related to the energy: We made the assumption that a light weight mobile sensor of 0.65 lb could travel at a constant speed of 2.5 cm/s.

### 4.3. Results and Discussion

A comparison has been made of the performance of the RIM and NN protocols and the proposed algorithm. For the experimentation, the distance travelled, number of exchanged messages, number of nodes moved, and percentage of the field coverage reduction were some of the parameters employed for measuring how well the proposed algorithm performed.

#### 4.3.1. Distance Moved

**Figure 4:**
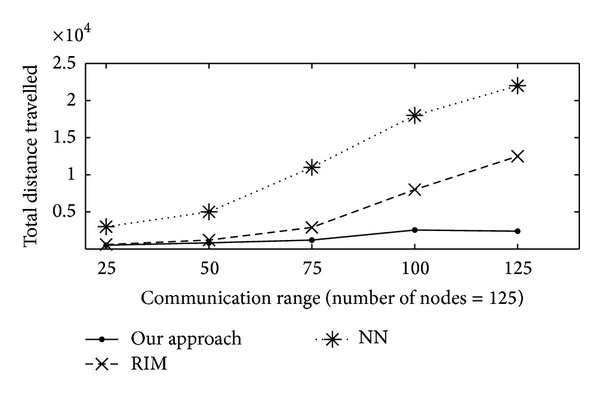
Total distance travelled by all nodes (meters) versus communication range (meters).

The total distance moved is measure of total distance moved by all nodes involved in the recovery which gauges the efficiency in terms of energy efficiency and overhead involved in the recovery. In Figure 4 the total distance moved is plotted on *y*-axis, with varying communication range (from 25 to 125 m) on *x*-axis. Figure 4 shows the total distance that nodes collectively had to travel during the recovery as a function of the communication range. Again, the sensing and communication ranges are equal in these sets of experiments. The distance a node would travel depends on the internode proximity, which is at most the communication range *r*_*c*_. This was easy in the cases of RIM and NN as the distance travelled grew at a high rate. Unlike RIM and NN, with our proposed algorithm, the node involvement was limited in the process of the recovery to only the neighbours of the node that failed. It did not make use of the cascaded relocation found in RIM and NN. It is worthy to mention that when rc was large, the proposed algorithm dealt with the rise in the connectivity of the network very well.

#### 4.3.2. Number of Exchanged Messages

**Figure 5:**
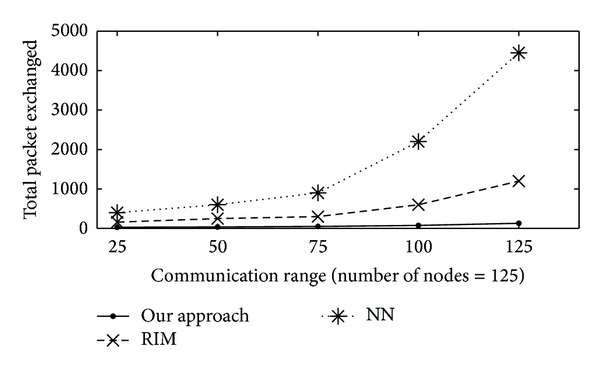
Total number of packets exchanged versus communication range (meters) for 25 to 125 nodes in the network.

Number of exchanged messages is measured by the number of messages exchanged among nodes. This is a measure of the recovery process overhead. In Figure 5, total packet exchanged is plotted on *y*-axis, with varying communication range (from 25 to 125 m) on *x*-axis. Figure 5 shows the total number of packets that were exchanged while restoring connectivity under all three methods. Each broadcast is counted as one message. The messaging overhead with proposed algorithm is minimal, while NN exchanges the most number of packets. This was because, in the proposed algorithm, only the failed node's neighbours were involved. On the other hand, with RIM and NN, the exchange of messages had to synchronise their action with all of the nodes that were repositioned. It is worth mentioning that the number of messages remained almost the same, in our proposed approach, even though there was an increase in the connectivity of the network for a large *r*_*c*_. This was because interaction rarely took place between the other concerned nodes and the coordinator. Again, Figure 5 presents results based on one round only although it would increase with time. On the other hand, the proposed algorithm can be scaled for several rounds. It would also provide connectivity and coverage restoration at a reasonable cost. This is quite a substantial advantage in performance over solutions that are permanent topology adjustment-based. All in all, our algorithm has been proven to impose only a little bit of messaging overhead as is seen in Figure 5. Moreover, it is suitable to be used with sensor nodes that are bandwidth constrained.

#### 4.3.3. Number of Moved Nodes

**Figure 6:**
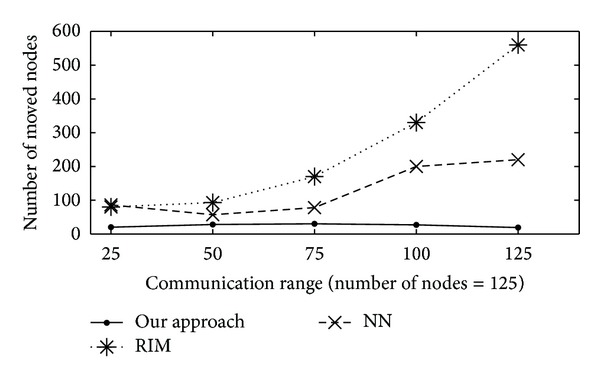
Total number of nodes moved versus communication range (meters) for 25 to 125 nodes in the network.

During the failure recovery the total number of moved nodes in all three baseline algorithm shown in Figure 6. Similar performance of proposed technique and RIM encountered as in manipulating of the total distance travelled by all nodes. For the reason that more the travelling of number of nodes, the total distance travelled will be higher. At this result the node movement of RIM is higher than NN. In any situation the less number of nodes movements is involved in proposed technique, because it limits the opportunity of recovery to only neighbouring nodes of the failed node.

#### 4.3.4. Percentage of Reduction in Field Coverage

**Figure 7:**
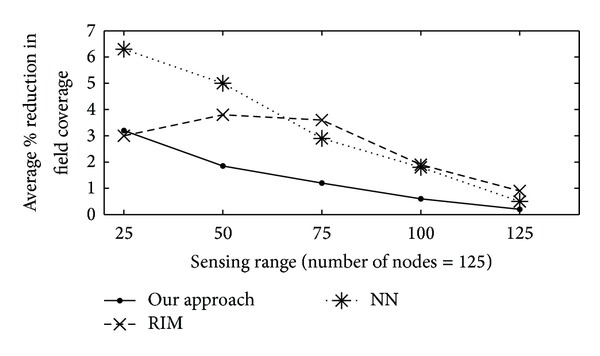
Reduction in field coverage versus sensing range (m) for 25 to 125 nodes.

**Figure 8:**
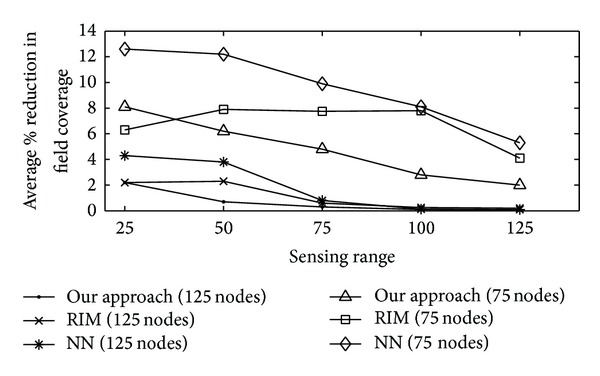
Reduction in field coverage versus sensing range (m) for 75 and 125 nodes.

**Figure 9:**
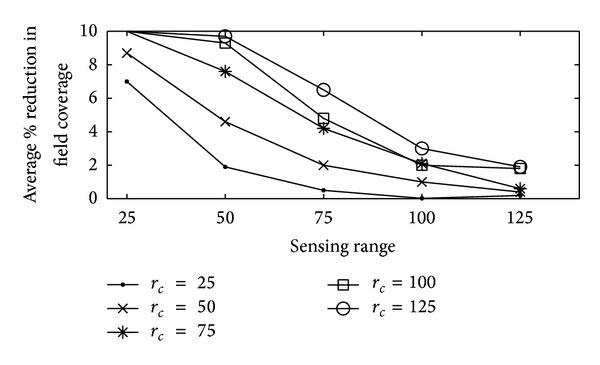
Reduction in field coverage for proposed technique versus sensing range (m) for different communication range (m).

Percentage of reduction in the field coverage is referred to as connectivity centric restoration impacts coverage, measured in terms of the percentage of reduction in field coverage relative to a prefailure level. In Figures 7–9, average percentage of reduction in the field coverage is plotted on *y*-axis, with varying number of sensor nodes (from 25 to 125) on *x*-axis. Figures 7 and 8 show how overall proposed algorithm could significantly limit the loss in coverage. For sparse networks where nodes are evenly distributed with minimal coverage overlap, the field coverage under proposed algorithm decreases by a similar amount to that in RIM. The field coverage level before the failure was sustained after our algorithm was applied. This was accomplished by the fact that, because of the increase in the coverage overlap, the replacement nodes needed only to move a short distance or not at all. Moreover, most of the home locations of the replacement nodes were still covered by other neighbour nodes when each node moved for its turn. Furthermore, there were several nodes available for the relocation process. However, networks with sparse node deployment did not have a lot of nodes that could be used to replace the node that failed. Further, a larger area was left unmonitored when the nodes were relocated. The result was a gap in the coverage of the network. The percentage of the field coverage reduction for various sensing and communication ranges is shown in Figure 9. Quite a bit of coverage reduction was noticed when *r*_*c*_ dominated *r*_*s*_. This was because longer distances needed to be travelled by the nodes between their home area and the position of the node they were replacing. Even so, the reduction was limited to 10% with the proposed algorithm even for *r*_*c*_ = 6 *r*_*s*_.

#### 4.3.5. Proposed Technique Summary of Results

This section discussed the simulations carried out in OMNeT++ to evaluate the performance of the proposed protocol and the results are compared against the contemporary baseline approaches. Simulation results demonstrate that proposed technique has achieved significant energy savings and enhanced the network lifetime. The results demonstrate that our approach is effective in improving QoS parameters, such as number of exchanged messages, average number of nodes moved, and percentage of reduction in field coverage, when compared with baseline approaches.  Table 3 summarizes the findings from this research work.

## 5. Conclusion

In mobile sensor networks, it is vital that the topology of the connected internodes is maintained. The network can be partitioned because of node failure and this in turn causes the operation of the application to be disrupted. Unlike most previous works that take advantage of the relocation of the nodes so that connectivity can be restored, the proposed algorithm deals with not only the connectivity loss issue but also the issue in the loss of the field coverage. In general, when there is no awareness of the coverage in the process of the resorting connectivity, there is a potential to cause some locations not monitored by any sensor. To solve this, the proposed algorithm does not relocate the nodes permanently. The recovery failure is the responsibility of the neighbouring nodes. The failed node's neighbours coordinate amongst themselves to decide on each of their roles in the process of the recovery. To restore connectivity, all of the neighbouring nodes taking part in the recovery process relocate themselves to the area of the failed node, one at a time, to provide coverage in that location. After spending an allotted amount of time replacing the failed node, each node returns to the location from which it started off. These neighbouring nodes take turns in this process. For a better application fit, for which the lifetime of the network is most valued, a balance between coverage and connectivity is provided by the proposed algorithm. It provides a balance of the load amongst the failed node's neighbours. The proposed method is a combination of algorithms that are localized and distributed. Messaging overhead is slight and the approach can be scaled for networks that are not small. Validation of the proposed method is achieved by simulation which has also proven the proposed method's effectiveness.

## Figures and Tables

**Algorithm 1:**
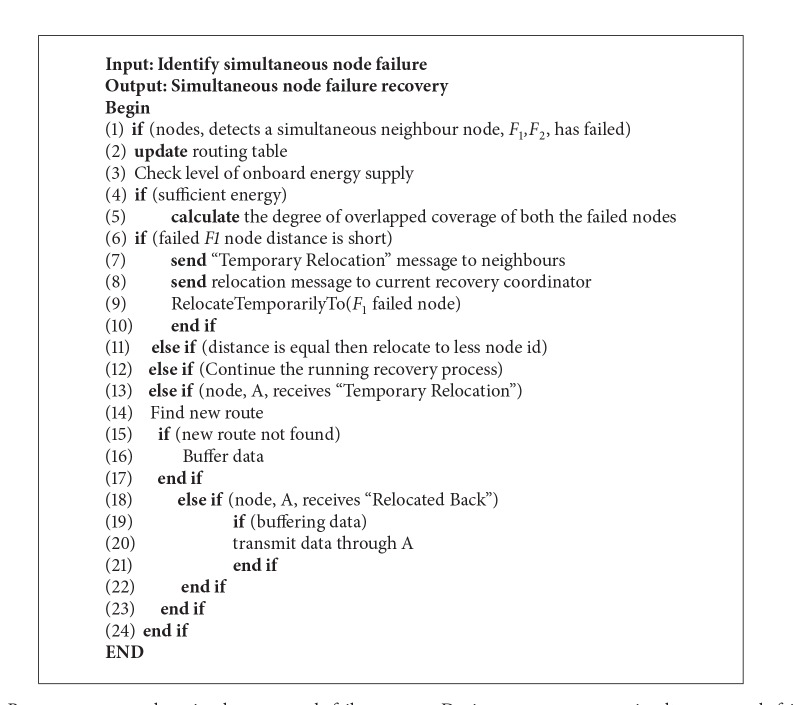
Recovery process when simultaneous node failure occurs. During recovery process simultaneous-node failure occurs.

**Table 1:** Protocol summary.

Reference	Optimization objective	Migration technique	Node type	Area partition	Network architecture	Sensor mobility	hops	Limitations/constraint
[28]	Connectivity restoration	Direct	Sensor	Voronoi diagram	Centralized	Robot	2	Convergence time: do not consider coverage
[29]	Connectivity restoration	Shifted	Data collector	Zone	Centralized	Robot	2	Convergence time: do not consider coverage
[31]	Coverage	Shifted	Sensor	Plane	Centralized	Actuator	2	Do not consider coverage
[32]	Connectivity	Shifted	Sensor	Square or hexagon	Centralized	Mobile sensor	2	Do not consider connectivity
[33]	Connectivity	Shifted	Data collector	Mesh	Distributed	Robot	1	Do not handle actor failure
[34]	Connectivity	Shifted	Data collector	Plane	Centralized	Actuator	2	Do not consider coverage
[35]	Connectivity	Shifted	Sensor	Plane	Centralized	Mobile sensor	2	Do not consider coverage
[36]	Coverage	Direct	Data collector	Plane	Distributed	Actuator	1	Consider neither coverage nor application level interests
[37]	Coverage	Shifted	Data collector	Plane	Distributed	Actuator	1	Do not handle actor failure
[38]	Connectivity	Shifted	Sensor	Grid	Distributed	Mobile sensor	2	Do not consider connectivity
[39]	Connectivity	Direct	Sensor	Grid	Distributed	Mobile sensor	2	Do not consider coverage
[22]	Connectivity and coverage	Direct	Sensors	Grid	Distributed	Mobile sensor	1	Do not consider multinode and simultaneous node failure

**Table 2:** Parameters for the communication energy model.

Term	Description
*β*_11_, *β*_12_	Energy dissipated in transmitter and receiver electronics per bit (take to be 25 nj/bit).
*β*_2_	Energy dissipated in transmitter amplifier (take to be 50 pJ/bit/m^2^).
*s*	Number of bits in the message.
*d*	Distance that the message traverses.

**Table 3:** Results summary.

Protocol compared (125 nodes)	Average travelled distance (m)	Average number of exchanged messages	Average number of moved nodes	Average reduction in field coverage (%)
Proposed technique	2200	200	30	3
RIM	12000	1900	150	11
NN	18000	3500	500	17
